# From concept to chemistry: integrating protection group strategy and reaction feasibility into non-natural amino acid synthesis planning

**DOI:** 10.1039/d5sc04898b

**Published:** 2025-09-08

**Authors:** Gökçe Geylan, Mikhail Kabeshov, Samuel Genheden, Christos Kannas, Thierry Kogej, Leonardo De Maria, Florian David, Ola Engkvist

**Affiliations:** a Molecular AI, Discovery Sciences, BioPharmaceuticals R&D, AstraZeneca Gothenburg Sweden gokcegeylan96@gmail.com; b Division of Systems and Synthetic Biology, Department of Life Sciences, Chalmers University of Technology Gothenburg Sweden; c Medicinal Chemistry, Research and Development, Respiratory & Immunology, BioPharmaceuticals R&D, AstraZeneca Gothenburg Sweden; d Department of Computer Science and Engineering, Chalmers University of Technology, University of Gothenburg Gothenburg Sweden

## Abstract

Incorporating non-natural amino acids (NNAAs) into peptides enhances therapeutic properties, including binding affinity, metabolic stability, and *in vivo* half-life time. The pursuit of novel NNAAs for improved peptide designs faces the challenge of effective synthesis of these building blocks as well as the entire peptide itself. Solid-Phase Peptide Synthesis (SPPS) is an essential technology for the automated assembly of peptides with NNAAs, necessitating careful protection for effective coupling of amino acids in the peptide chain. This process requires orthogonal protection of the reactive groups in individual amino acids after synthesizing them, presenting a challenge in bridging *in silico* peptide design with chemical synthesis. To address this, we have developed a first-of-its-kind synthesis assistance tool, NNAA-Synth, that plans and evaluates the synthesis of individual SPPS-compatible NNAAs. Our tool unifies (i) introducing orthogonal protecting groups to NNAAs, (ii) retrosynthetic prediction to propose synthesis routes, and (iii) scoring the synthetic feasibility of these routes. We demonstrate how the tool facilitates optimal protection strategy selection for individual NNAAs. Additionally, it enables synthesizability-aware NNAA ranking and prioritization during computational screening, enhancing the quality of the *in silico* design by assessing the accessibility of individual building blocks.

## Abstract 2

NNAA-Synth is a synthesis assistance tool for SPPS-compatible non-natural amino acids that integrates protection strategies and evaluates reaction feasibility.

## Graphical abstract

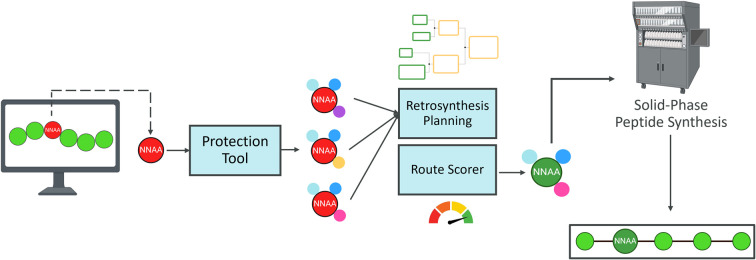

## Introduction

The search for novel therapeutic agents in drug discovery has expanded beyond small molecules, with peptide therapeutics established as a promising modality.^1–3^ Peptides have gained importance for their ability to target large or shallow clefts previously deemed to be undruggable by small molecules harnessing the potential to modulate protein–protein interactions.^4^ Peptides exhibit desirable drug-like properties such as high specificity and low toxicity; however, design challenges remain in terms of metabolic stability, oral bioavailability, and permeability among other properties.^5,6^ For the protein space, enzymes that are efficient biocatalysts, signaling molecules that regulate biochemical cascades, antibodies that induce immunogenicity, and many other therapeutic agents are being explored.^1,7,8^ Properties such as high specificity, stability, and potency, similar to the peptide-related challenges, are critical considerations in protein engineering projects.^7,8^

To meet these design requirements, it is essential to explore the peptide or protein chemical space exhaustively. The traditional sequence space is vast due to the combinatorial possibilities of the proteinogenic building blocks composed of 20 natural amino acids.^7^ Although the large sequence space can offer potential drug candidates for molecule optimization, enhancing especially the peptide properties heavily relies on the incorporation of non-natural amino acids (NNAAs) in drug discovery and development projects.^5^ NNAAs expand the chemical diversity of peptides to a space theoretically similar to that of small molecules with unique functional groups, varying sidechains, and the modifications in the backbone.^9^ Common modifications to natural amino acids such as backbone *N*-methylation or stereocenter inversion are known to improve the oral bioavailability and permeability of peptidic drugs.^10^ More complex amino acid designs such as novel sidechains that introduce hydrogen bonding between sidechain and backbone have been incorporated into peptides to improve the passive diffusion of peptides through cell membranes and to enhance their solubility.^10,11^ Recently, these design strategies have been explored by generative models. We have recently developed PepINVENT, a generative model for peptide design.^12^ PepINVENT navigates the vast space of natural and non-natural amino acids to propose valid, novel, and diverse peptide designs.^12^

The novel designs and complex derivatives of commercially available amino acids introduce synthesis challenges when considering NNAAs for peptide optimization. To bridge *in silico* designs to wet lab, a careful consideration of synthesis planning is required. Solid-phase peptide synthesis (SPPS) is an efficient method that enables sequential addition of amino acids to a growing peptide chain anchored to a solid support.^13^ A peptide containing one or more NNAAs can be chemically synthesized using SPPS techniques, provided all the amino acids are available.^13^ The reactive fragments in the building blocks or the amino acids of the peptide of interest need to be protected before being incorporated to the SPPS process. The amino acids must be protected with orthogonal groups between the backbone and sidechain, to ensure the correct assembly of the peptide chain.^13,14^ During the peptide synthesis, the protection groups are selectively removed and used to elongate the chain in the correct sequence by coupling reaction. The cycle of deprotection and coupling continues until the peptide sequence is completed and the process is finalized with the removal of all protection groups from the chain.^15^ This deprotection and coupling strategy minimizes side reactions by ensuring the addition of amino acids to specific positions and with specific connectivity to the peptide chain. SPPS facilitates controlled synthesis of peptides and the protected versions of many natural and NNAAs are commercially available from vendors, ready to be plugged in to SPPS.^13^ However, any novel NNAA, not readily available needs to be chemically synthesized and orthogonally protected in order to be compatible with SPPS. The choice of the protection strategies for the reactive groups in NNAAs can influence the yield of the SPPS-ready building block as well as the coupling reaction during production.^15^

Synthetic feasibility of novel and protected NNAAs provides a significant challenge with complex synthetic routes, availability of the starting materials, and the protection strategy. To address these challenges, we introduce a novel cheminformatics tool, NNAA-Synth, designed to evaluate the synthesizability of protected α-NNAAs. Our tool uniquely aims to provide insights into the synthetic feasibility of the protected amino acids through the optimal protection strategies, retrosynthetic planning of the NNAAs and deep learning (DL)-based feasibility scoring. By approaching the chemical synthesis and protection challenges of NNAAs as a single, integrated problem, NNAA-Synth provides synthesis solutions to streamline the SPPS-compatible amino acid production and to inform the design process with building block accessibility. To our knowledge, no existing solution combines the protection, synthesis planning and feasibility assessment into an all-in-one tool for NNAA design and synthesis planning. In this paper, we will demonstrate potential use cases for our tool with respect to (i) selecting the most synthetically feasible NNAA from a set of options to help the medicinal chemists design drug candidates, and (ii) choosing the optimal protection strategy for a novel NNAA for efficient incorporation into peptide synthesis.

## Methods

### Amino acid data

A set of unprotected α-amino acids was obtained from Amarasinghe *et al.*^9^ The dataset was created as a large virtual library from eMolecules database.^16^ Following a reaction-based enumeration method, a set of 380 K NNAAs were generated from reagents that can yield readily synthesizable NNAAs using Strecker, Gabriel or amination synthesis approaches.^9^ It is worth noting that no reaction feasibility assessment was done during the enumeration apart from ensuring that a single reactive function was present in every selected building block. Subsequently, a chemically diverse set of 9985 α-amino acids made publicly available by this study, was employed for the development of our tool.^9^

### Identifying reactive groups and deriving heuristics for protection

**Fig. 1:**
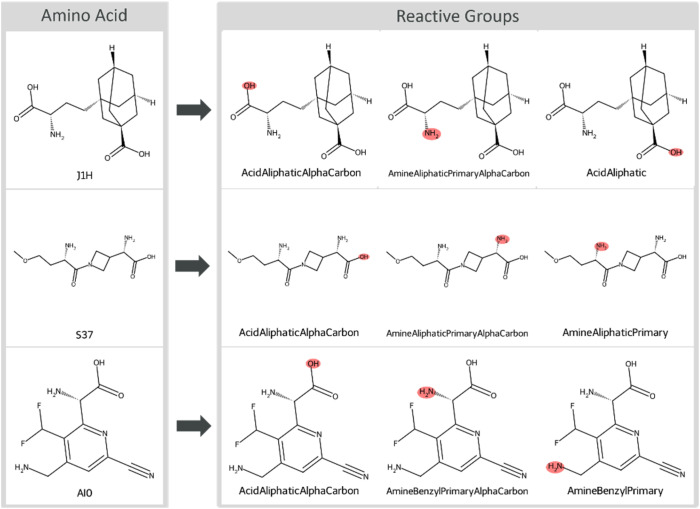
Depiction of three NNAAs with their respective reactive groups annotated. The amino acids are labeled with their names as specified in Amarasinghe *et al.*^9^

Reference heuristic containing a comprehensive library of SMILES Arbitrary Target Specification (SMARTS) patterns to represent specific reactive groups within molecular structures was utilized (Table S1). The heuristics were used to systematically scan the input molecules against the reference SMARTS, detecting the matching patterns. The reactive groups corresponding to the matched patterns were identified as the substructures that need to be protected in the diverse set of 9985 amino acids. The heuristics were extended to separately capture the sidechain and backbone reactive groups, enabling the orthogonal protection strategies in later steps. The extension contained separate SMARTS pattern defining the backbone connectivity for (i) aliphatic acids for the carboxylic acid in the backbone, (ii) primary aliphatic amines, and (iii) primary benzyl amines for the amino group in the backbone (Fig. 1).

Each reactive group identified by the SMARTS patterns was associated with one or more protection groups (Fig. S1). To obtain broad coverage of the large chemical space of α-amino acids, four classes of mutually orthogonal protecting group were combined (Table S2), each can be cleaved by a distinct method: acid, base, hydrogenation, oxidation or fluoride. This strategy enables stepwise, independent exposure of the desired functional groups without protecting group interference during deprotection. In the amino acid backbone, carboxyl termini were masked with *tert*-butyl esters (*t*Bu), which are rapidly released by strong acids such as trifluoroacetic acid. In contrast, amino termini was protected with fluorenylmethoxycarbonyl (Fmoc) carbamate, that can be selectively removed under basic, non-nucleophilic conditions, with piperidine.^15,17^ This mirrors the classical Fmoc/*t*Bu regime used in SPPS yet remains compatible with solution-phase couplings.^17,18^ In the sidechain, a range of protection groups was introduced to allow for selective deprotection under conditions orthogonal to those used for deprotecting the backbone. Benzyl-based groups (benzyl (Bn) for acids/alcohols and 2-chlorobenzyloxycarbonyl (2ClZ) for amines) are removed by hydrogenolysis, making them entirely orthogonal to both acid and base lability and stable to most peptide coupling reagents.^17^ For sidechain hydroxyls or thiols must survive the foregoing manipulations, oxidatively labile *p*-methoxybenzyl (PMB) ethers and sulfides are introduced. These can be cleanly detached with DDQ, leaving all benzyl-type protections untouched.^17^ Finally, the trimethylsilyl-ethyl (TMSE) esters and ethers, when used to protect sidechain acids or alcohols, can be selectively removed with tetrabutylammonium fluoride (TBAF), while withstanding acid, base, hydrogenation and oxidative conditions. Overall, strategic permutation of these protecting group classes grants a controlled deprotection sequence over the assembly and modification of complex peptides and accommodating diverse sidechain functionalities.^15,19,20^ This approach enables late-stage diversification of densely functionalized NNAA scaffolds while preserving sensitive γ- or δ-heteroatoms. For example, the backbone of an amino acid protected by Fmoc/*t*Bu while the sidechain reactive moieties by Bn/2ClZ, PMB, TMSE can go under deprotection order of base → hydrogenolysis by H_2_/Pd → oxidation by DDQ → fluoride-mediated cleavage by TBAF, respectively (Table S2). The suggested algorithm follows the principles articulated in the protecting group monographs of Greene and Wuts and of Kocienski, and it has been exploited recently in modular syntheses of non-canonical residues for ribosomal incorporation, peptidomimetics and macrocycle design.^17,19–24^

### Reaction template construction

**Fig. 2:**
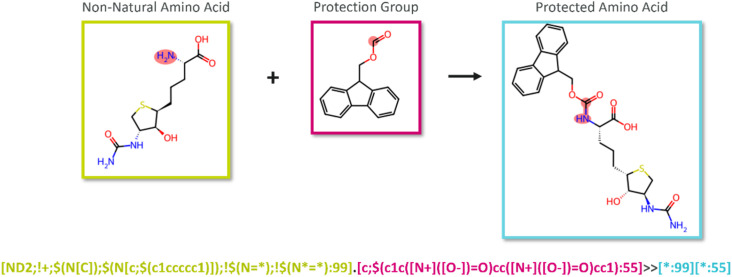
Schematic description of the generation of reaction templates to protect the reactive substructures of NNAAs. The reaction SMARTS pattern defines the reactive group atoms of the NNAA (green) and the protection group (pink) that will be linked to produce a protected NNAA (blue). These atoms are indexed by arbitrary numbers, 55 and 99, to ensure correct alignment during the cheminformatics reaction. This specific reaction pattern illustrates the attachment process where the nitrogen atom of the aliphatic primary amine in the backbone connects with Fmoc.

The identified reactive groups, along with the corresponding protection strategies, were encoded into reaction templates using the associated SMARTS patterns. These templates were constructed as reaction SMARTS to be utilized by reaction functionality of RDKit v.2023.9.6 (Fig. 2). This approach enabled the systemic protection of the input amino acid with the assigned protection strategies.

### Protected series for amino acids

**Fig. 3:**
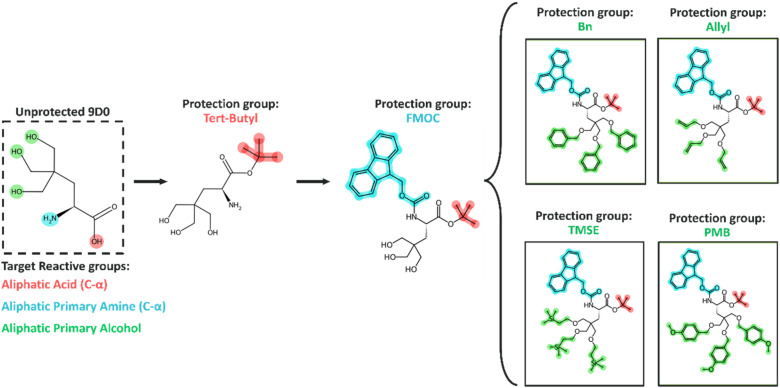
The illustration shows the selective protection of reactive groups in an unprotected NNAA. Protection is achieved through recursively passing the NNAA as a reactant to a list of reaction templates. This list was constructed from the reactive substructure mapping to potential protection groups and the corresponding reaction template. Generating the protected NNAA with all possible protection strategies yields a set of protected forms. This specific example highlights an unprotected amino acid, 9D0, with identified reactive groups, and with each protection group shown in matching colors.

The library of NNAAs was protected using the reaction templates by introducing the amino acid and its protection group as reactants through methods in RDKit v.2023.9.6.^25^ For each amino acid, reactive substructures were sequentially protected by using the product of one reaction as the reactant for the next. This iterative process ensured the protection of all identified substructures. In cases where the reactive substructures were assigned with multiple protection strategies, each protection group was applied separately, resulting in multiple protected versions of a given NNAA (Fig. 3).

### Retrosynthesis planning

The retrosynthetic planning was carried out using AiZynthFinder software.^26,27^ The software conducts a retrosynthetic analysis for a target molecule by utilizing Monte Carlo Tree Search (MCTS) algorithm enhanced by neural networks.^26^ The target molecule is recursively broken down into readily available starting materials by the search algorithm. Neural networks guide the route search to optimize the potential starting materials selection using the known reaction templates.^26^

The library of NNAAs, protected with various strategies, were input to AiZynthFinder that returns the predicted synthetic routes. Expansion and filter models trained on data from the United States Patent and Trademark Office (USPTO)^28^ were used. Publicly available eMolecules building block set^16^ and the selected protection groups were supplied as the library of purchasable starting materials. The maximum search depth was set to 15 to generate a comprehensive exploration of potential synthetic routes. A key aspect of the runs involved applying a filter strategy to restrict the decomposition of bonds in the extensive protective groups with complex substructures such as TBDMS, Fmoc, DNP, TMSE, *t*BuS, Tos, DPSide. Freezing the protection groups as substructures enables them to be included into the predicted routes as purchasable starting material, rather than requiring synthesis from scratch. The remaining parameters were set to their default values. All reactions in the proposed routes were annotated with their reaction classes using the NameRxn software.^29^

### Scoring the synthetic feasibility

The output of AiZynthFinder contains routes for each input molecule, protected amino acids in our case. The proposed routes were subjected to route scoring algorithms of Chemformer-based feasibility filtering^30^ followed by an expert-augmented DL model^31^ to assess the feasibility of the routes.

Chemformer, a transformer-based model pre-trained on SMILES, was fine-tuned for both product prediction from reactants (forward Chemformer) and reactant prediction from products (backward Chemformer) as forward Chemformer using 18.7 million public and AstraZeneca's proprietary reactions.^30,32^ These models were used to assess round-trip accuracy by measuring the consistency between forward and backward predictions in retrosynthetic analysis. Demonstrating high round-trip accuracy in both single-step (above 0.97) and multistep retrosynthesis with unseen reactions, it is suggested for use in a mixed-policy approach with the template-based AiZynthFinder for route evaluation.^30^ The product prediction, or the forward Chemformer, was utilized as a feasibility filter that excludes chemically implausible transformations by generating a predictive probability-focused score for individual reactant(s)-product pairs in a route.^30^ Each score was calculated by predicting the product at each step in the retrosynthetic tree, using the corresponding reactants provided by AiZynthFinder as input.^30^ For chlorination and bromination reactions, the output from AiZynthFinder was augmented with either chlorine or bromine, respectively if it was missing because not all retrosynthesis templates generate the halogenation reagent. Chemformer, with a beam size of 10, predicted a set of potential products for the given reactants and assesses whether the true product, in the AiZynthFinder prediction, appeared in this batch.^30^ If found, the probability of the prediction became the score; if not, the reaction was assigned a score of zero. Subsequently, scores across all steps in a route were aggregated into a single score by multiplying them. Routes with a feasibility score greater than zero were proceeded to the second round of assessment, while those scoring zero were considered synthetically unfeasible and excluded from further analysis.

The second and final route scorer was another DL method informed with chemists' expert assessment on multi-step synthesis routes by Guo *et al.*^31^ The expert augmented method provides an overall assessment of route feasibility by combining reaction-level, route-level, and target molecule-based descriptors. This model utilized 47 303 historical synthetic routes from Journal of Medicinal Chemistry as reference routes and AiZynthFinder-generated routes for the same target molecules as proposed routes.^31^ The model was trained to predict the distance between proposed and reference routes based on embeddings of three features: (i) route, *i.e.* cost, reaction complexity, and precursor availability, (ii) reaction, *i.e.* a statistical assessment of the reaction feasibility of propriety data and structural difference fingerprint encodings, and, (iii) the target molecule represented with Morgan fingerprints.^31^ The distance-based scoring was further refined into an augmented score by incorporating route length with weights determined by fitting the scores to expert insights on feasibility for a subset of proposed routes.^31^ The resulting score was shown to agree with the expert opinion, achieving a Pearson correlation coefficient of 0.92 and therefore used as a proxy for the expert opinion.^31^ This score was then used as the route quality, or feasibility score and was categorized into “Good”, “Plausible”, and “Bad” routes based on ratings from the experts:^31^
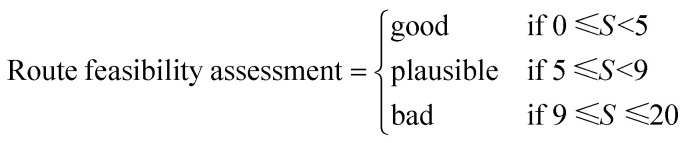
where *S* represents the route feasibility score output from the neural network.^31^ The optimal route for a given target was chosen based on the lowest expert-augmented feasibility score, also referred as Synthesis Feasibility (SF) score in this study.

## Results

In the results section, we provide analysis on the series of cheminformatics, retrosynthesis planning and route evaluation steps that make up our tool as well as demonstrating how the tool can be utilized (i) to select the optimal protection strategy for a given amino acid, and (ii) in an *in silico* drug discovery project to select a candidate NNAA for mutating a therapeutic peptide to improve its binding affinity.

### Identification of the reactive substructures and their protection strategies

**Fig. 4:**
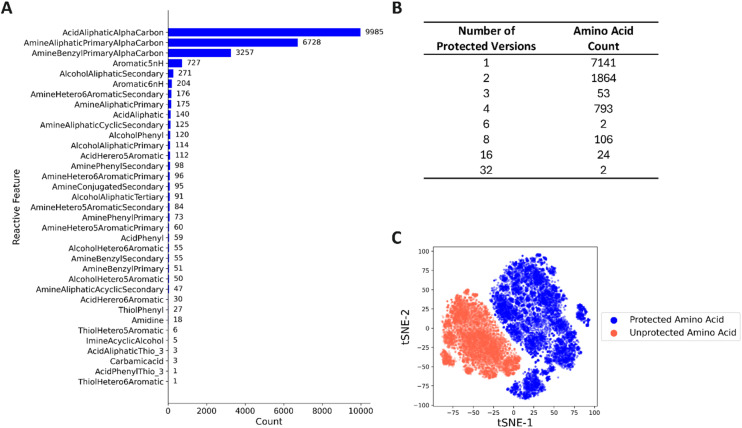
The diversity of the reactive substructures and the resulting impact of potential protection strategies on the expansion of the chemical space considered for the retrosynthetic challenge was described. (A) The distribution of the reactive groups among the library of NNAAs were shown. The top three reactive features define the carboxylic acid and the aliphatic or benzylic amino group present in every α-amino acid backbone. (B) The number of amino acids and their corresponding protected versions, ranging from 1 to 32 distinct molecules produced from the combinatorial protection, are shown. (C) The chemical space was visualized through t-distributed Stochastic Neighbor Embedding (t-SNE) on 512 bit count-based Morgan fingerprints with radius = 3, usechirality = true parameters.

The NNAA library with diverse sidechains necessitates the identification of reactive functional groups to be protected for the chemical synthesis with SPPS. The reference heuristics encompassing all the reactive substructures present in our library was constructed with SMARTS patterns. The substructures and their distributions within the library illustrate the diversity of reactive groups among the sidechains (Fig. 4A). The top three structures featuring “AlphaCarbon” represent the N- and C-terminals of α-amino acid backbone, while the remaining 32 groups characterize the reactivity of the sidechains (Fig. 4A).

Following the reactive group annotation, the NNAAs were appropriately protected using a custom mapping of reactive functional groups to protection groups. As most amino acids lacked reactive functional groups in their sidechains (approximately 7000 of them), the protected building blocks consisted of primarily backbone-decorated molecules. In contrast, the remainder of the library, around 3000 NNAAs, required sidechain protection, and was subjected to multiple strategies, generating a protected series of the individual amino acids (Fig. 4B). 9985 NNAAs were expanded into 15 508 protected residues for retrosynthesis planning.

### Understanding the complexity of the synthesis challenge

Starting with a diverse NNAA library, the synthesis challenge can be regarded as similar to synthesizing a set of small molecules. However, the true complexity of the problem becomes apparent when considering the protection strategies required for SPPS, highlighting that the synthesis of individual unprotected amino acids is insufficient. Analysis of the chemical space of both unprotected and protected NNAA libraries reveals that introducing the protective groups increases the molecular complexity of individual NNAAs (Fig. S2). This, in turn, expands the overall size of the chemical space that must be explored for synthetic accessibility, making the synthesis challenge significantly more complex (Fig. 4C and S2).

### Evaluation of retrosynthetic routes and their synthetic feasibility

The retrosynthetic planning for protected NNAAs, proposed by AiZynthFinder, generates multiple synthetic routes for each target molecule. A comprehensive evaluation of these routes focusing on some key metrics such as the availability of the suggested precursors, number of reactions in each route, and the state score for each target molecule were explored. Additionally, both feasibility scores calculated in this study were analyzed for the NNAA library. These metrics are shown for the most feasible routes for each NNAA (Table 1) and the entire protected NNAA library, separately (Fig. S3).

**Table 1:** The metrics describing the number of molecules explored with the features of routes proposed by AiZynthFinder. Additionally, the table shows the distributions of the two feasibility scoring methods employed in this study. The protected NNAAs describes the most feasible routes selected for the protected NNAA library while NNAAs with optimal protection refers to the remaining routes after selecting the most feasible protection strategy for each amino acid. All route metrics are provided as the mean ± standard deviation format with corresponding distributions provided in Fig. S3

Metrics	Protected NNAAs	NNAAs with optimal protection
Total number of molecules	15 508	9985
Infeasible molecules	4435	1692
Starting material availability	89.26 ± 10.40	89.19 ± 11.27
Number of reactions	8.95 ± 4.92	8.60 ± 4.98
Chemformer-based feasibility score	0.05 ± 0.15	0.07 ± 0.16
Expert-augmented feasibility score	8.63 ± 5.48	7.86 ± 5.40

While the entire protected NNAA library and the subset of this library encompassing the NNAAs with their optimal protection strategy exhibit similar distributions in route composition, the expert-augmented feasibility score, demonstrates better outcomes for the subset, as reflected by lower scores (Table 1). This suggests that the synthetic challenge, influenced by the increased molecular complexity from building block protection, was reflected not directly in the route composition but in the ultimate multi-parameter feasibility assessment of individual reactions. The multi-parameter scoring system emphasized route quality by considering, but not prioritizing, factors like route length, reaction types or the starting material availability. As a result, 40% of the protected NNAAs had routes with fewer than four reactions, compared to 33% of the individual NNAAs for which such routes were proposed. In contrast, when assessing the most feasible routes for protected molecules, 20% were classified as “solved”, *i.e.* all starting materials were commercially available, compared to 25% for individual NNAAs, demonstrating an inverse relationship.

Finally, 4435 protected molecules were assessed to be completely synthetically infeasible, corresponding to 1692 of the NNAAs that cannot be synthesized in the protected form (Table 1). These infeasible NNAAs can thus be excluded when conducting *in silico* screening methods for a better candidate selection.

### Application examples

In this section, we present potential application examples of our tool, including (i) a scenario in which we demonstrate how to adequately protect an amino acid of interest and propose a synthesis route that maximizes its feasibility; and (ii) leveraging the synthetic feasibility assessment to select NNAAs from a given set of options in a computational screening context. The examples aim to showcase use cases of our tool that can effectively connect *in silico* designs to potential wet-lab experiments.

### Strategic consideration for individual NNAA protection and retrosynthesis planning with feasibility assessment

When an NNAA is planned for incorporation into a peptide of interest, it, along with the other amino acids comprising the peptide, are protected and placed into SPPS as building blocks. Although commonly used amino acids such as the natural and standard non-natural ones are already purchasable from vendors in protected forms, a novel NNAA still necessitates the protection to make it compatible with SPPS. This example considers the protection challenge brought by the aim of chemical synthesis of an NNAA of interest.

**Fig. 5:**
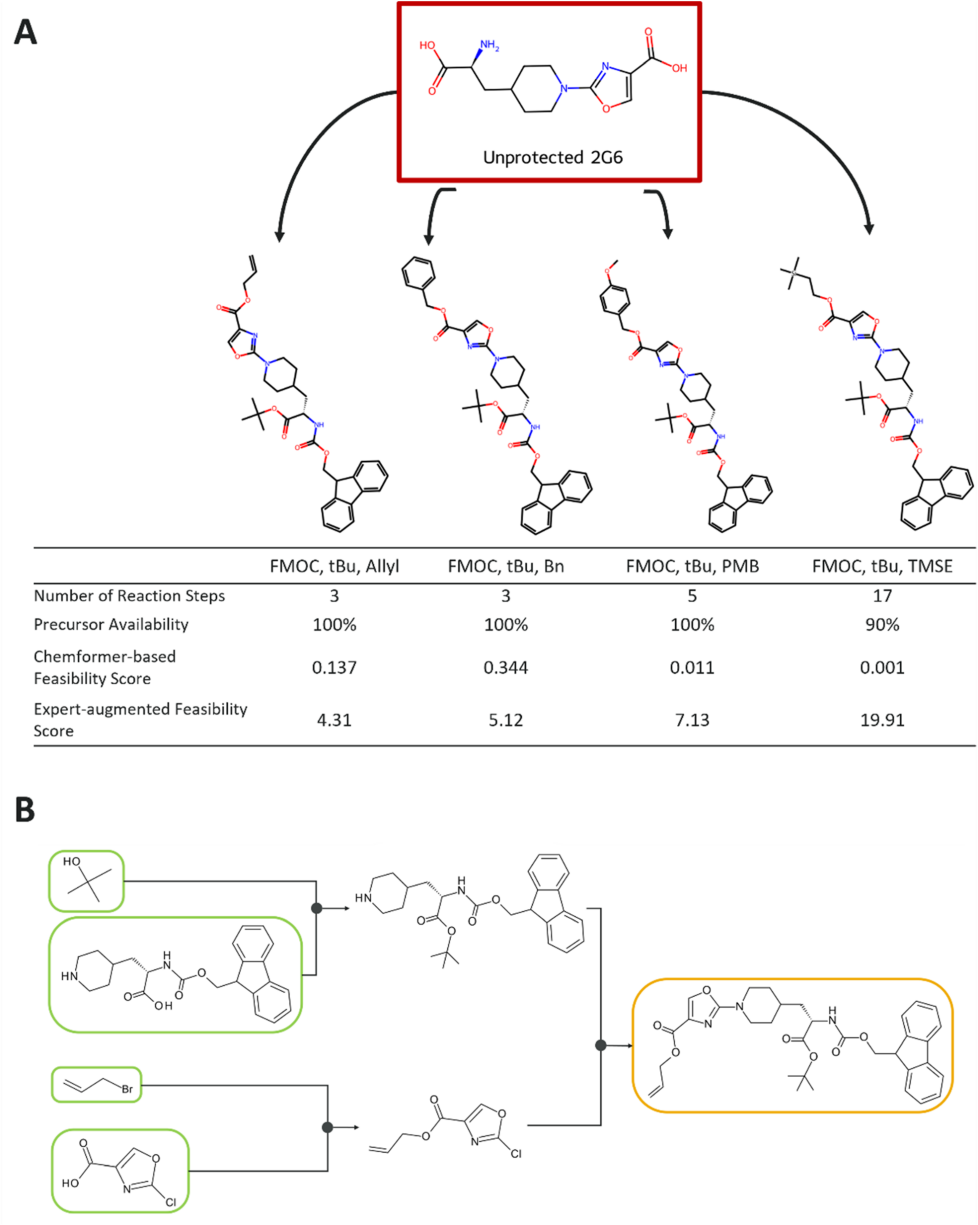
The selection of the most feasible protection strategy for an NNAA is shown. (A) The example showing the unprotected amino acid, 2G6, alongside the potential protection strategies that could be applied. The protected forms are annotated with their added protection groups, precursor availability, synthetic feasibility scores from both scoring methods, and the number of steps in the best proposed route. (B) The proposed route with the highest feasibility score for the selected protected form, (Fmoc, *t*Bu, allyl), is displayed. The starting materials are outlined with a green frame if they are available in the provided stocks, and the target molecule is outlined with a yellow frame. The routes for the remaining protected NNAAs can be found in Fig. S4.

The NNAA amino acid, named 2G6, demonstrates how the transition from NNAA to SPPS-compatible NNAA can be made through the synthesizability assessment. 2G6 can be protected through four different set of protection groups (Fig. 5A). NNAA-Synth uses the components in its pipeline to protect the NNAA and later to rank the synthetic feasibility (SF) score of the protection strategies. One of the protection strategies, (Fmoc, *t*Bu, TMSE), was deemed infeasible, as its route receiving the highest value for the expert-augmented feasibility, or the SF score of 19.91 and was not solved to commercially available starting materials (Fig. 5A and S4). Two of the protection strategies, with protection sets of (Fmoc, *t*Bu, Bn) and (Fmoc, *t*Bu, PMB), had moderate feasibility with score 5.12 and 7.13, respectively (Fig. 5A and S4). Finally, protecting the carboxylic and amine group in the backbone with Fmoc and *t*Bu, respectively and the heteroaromatic acid of a five-membered ring in the sidechain with allyl was the most synthetically feasible strategy for 2G6, achieving the lowest SF score (Fig. 5A). The score was also below 5 indicating “good” feasibility. Although 2G6 with (Fmoc, *t*Bu, Bn) and (Fmoc, *t*Bu, allyl) contained 3-step synthesis with all starting materials available in the eMolecules stocks, the SF score enabled the distinction of route qualities between them. In addition, all three steps of the best suggested route-esterification, carboxylic acid allylation and Buchwald–Hartwig cross-coupling-belong to the established, broadly used reaction classes and very similar to the successful syntheses reported in the literature.^33–35^ The routes with moderate feasibility, despite having feasible disconnection strategy, have less optimal reactants and issues with regioselectivity. The proposed route could then be followed through to synthesize the NNAA and to incorporate it to the building block library of SPPS (Fig. 5B).

### Decision-making for synthesizing a set of NNAAs derived from *in silico* screening campaigns

The second application example aims to demonstrate *in silico* selection of candidate NNAAs where synthetic feasibility serves as an additional criterion to improve the efficiency of design-to-make translation in Design-Make-Test-Analyze (DMTA) cycles. A model study from Amarasinghe *et al.*^9^ that uses peptide-protein complex from the Keap1–Nrf2 interaction as a model system was adapted. Kelch-like ECH-associated protein 1 (Keap1) binds to and consequently regulates the nuclear factor erythroid 2-related factor 2 (Nrf2) activity to combat oxidative stress.^36^ In normal conditions, with low levels of reactive oxygen species, Keap1 targets Nrf2 for degradation, preventing it from promoting gene expression related to the synthesis of antioxidant enzymes.^9,36^ During stress, Nrf2 translocates to nucleus to activate the stress response signaling.^9,36^ Keap1-regulated Nrf2 has been extensively researched and targeted in clinical studies for potential treatments of cancer as well as autoimmune, metabolic and neurodegenerative disorders.^36^

**Fig. 6:**
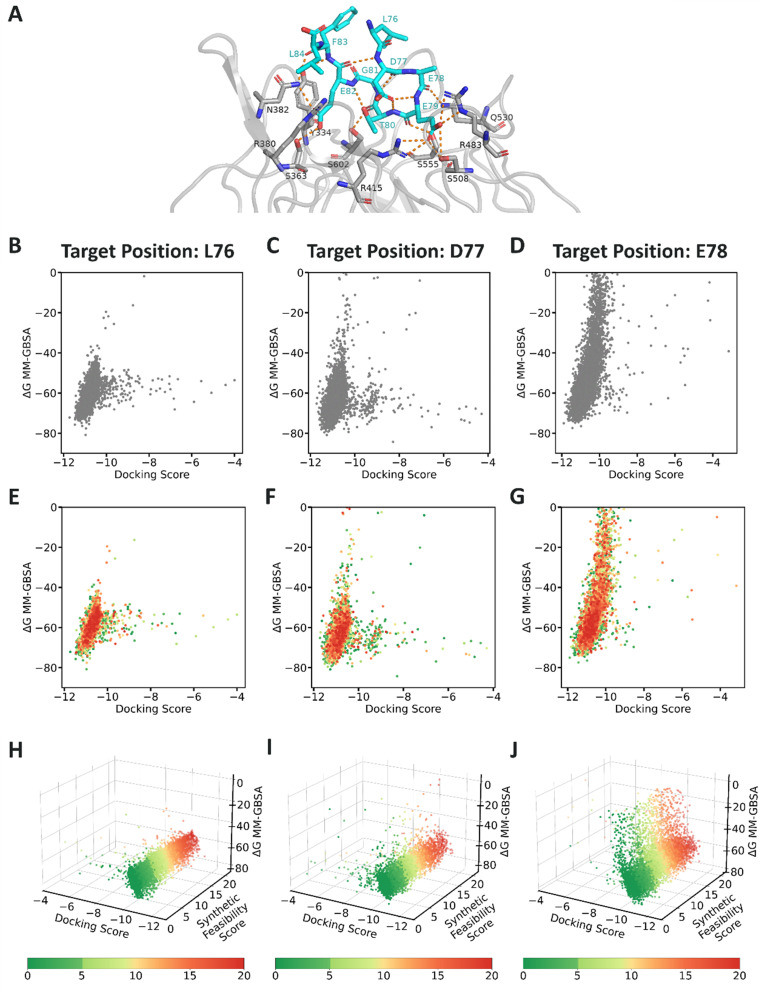
(A) The binding site of Keap1-Neh2 domain of Nrf2 structure. The complex was adapted from the Keap1-PDB ID: 2FLU.^39^ The VS experiment informed by synthetic feasibility of the screened NNAAs for the three docked positions were visualized for L76 in (B, E, H), D77 in (C, F, I), and E78 in (D, G, J). (B–D) The docking scores of NNAAs plotted against Δ*G* MM-GBSA, (E–G) the same scores colored by the synthetic feasibility scores, and (H–J) all three scores by projecting the synthetic feasibility scores to the third dimension are visualized for each position.

A 16-mer peptide derived from Nrf2, containing the ^77^DxETGE^82^ motif within the Neh2 domain, was identified for its high binding affinity to Keap1 with a dissociation constant (*K*^solution^_D_) of 23.9 nM.^37,38^ Subsequently, a 9-mer peptide, isolated from the 16-mer peptide, was shown as the minimal peptide sequence mimicking the binding interaction of Nrf2 with Keap1 (Fig. 6A).^37^ The 9-mer peptide, with the sequence of ^76^LDEETGEFL^84^, exhibited moderate affinity to Keap1 with *K*^solution^_D_ of 352 nM.^37^

Amarasinghe *et al.*^9^ focused on enhancing the binding affinity of the 9-mer peptide through a large-scale virtual screening (VS) campaign. They conducted VS by mutating the first three positions (Leu76, Asp77, and Glu78) of the peptide of the peptide-Keap1 complex (PDB ID: 2FLU).^9,39^ Each residue was individually mutated to all other natural amino acids as well as to the library of 10 000 NNAAs, which also served as the foundational data for our study.^9^ Docking and molecular mechanics generalized born surface area (MM-GBSA) scores were calculated for each mutated peptide (Fig. 6B–D).^9^ NNAAs with superior docking scores compared to natural counterparts were identified as candidates for affinity improvement. While the study establishes an enumerated NNAA library to enhance peptide binding affinity, it does not investigate the practical synthesizability of these candidates for wet lab applications.

In this study, we extend this peptide site-specific mutagenesis study to include synthetic planning assessment (Fig. 6E–G). The SF score generated by our tool was integrated as an additional component in selecting candidate NNAAs (Fig. 6H–J). This incorporation allows for the selection of the NNAAs to potentially improve the peptide binding, informed by the synthesizability. The majority of the successfully docked residues showed “good” feasibility when protected according to the SF score (Table S3). Although the NNAA library was enumerated from eMolecules,^16^ approximately 30% of the docked NNAAs were assessed to be not SPPS-compatible as none of the potential protection strategies were deemed to be synthetically feasible.

**Fig. 7:**
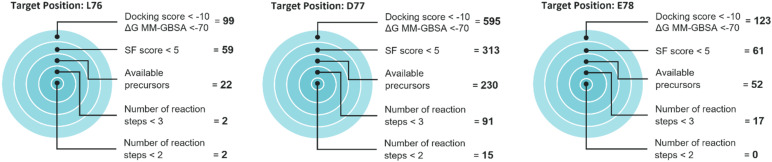
The target plots showcase the positions selected for peptide mutagenesis, with the number of NNAAs exhibiting favorable docking and Δ*G* MM-GBSA scores in the VS screening and the corresponding breakdown of their synthesizability. Upon evaluation with our tool, those assessed as synthetically feasible were further shortlisted based on the proposed synthetic routes, considering available starting precursors and the number of reactions involved in the route.

Automated synthesis planning enables the assessment of proposed routes for the NNAAs chosen for wet lab testing to potentially improve peptide-Keap1 binding. Key qualities evaluated include the SF score, availability of feasible route precursors in stock, and the number of reaction steps required to complete the synthesis. Considering these features, the synthesizability of the most optimal protection strategy was selected for individual building blocks. Among the selected protected NNAAs, there are 2, 91 and 17 of them were synthetically suitable, with less than 3 reaction steps, to mutate our peptide of interest in positions Leu76, Asp77, and Glu78, respectively (Fig. 7). Moreover, 2, 15, and 0 NNAAs of those were either already in the stocks or can be synthesized with a single reaction step from a similar starting material in stocks for positions Leu76, Asp77, and Glu78, respectively (Fig. 7). Asp77 was shown to be the most mutatable position with highest number of synthesizable NNAAs with favorable docking scores. Consequently, the score can be utilized to rank NNAA candidates as well as to prioritize a mutation site in VS as a synthetically accessible position.

## Discussion

In this paper, we introduce a novel computational tool, NNAA-Synth, to assist with synthesizability of NNAAs by unifying cheminformatics, established retrosynthetic planning and DL-based scoring methods. NNAA-Synth provides a practical solution to efficiently connect the computational peptide design to peptide synthesis and experimental validation. It addresses the challenges associated with incorporating NNAAs into peptides, an essential step when designing peptides to improve the therapeutic properties of drug candidates. By offering a synthetic feasibility score for each NNAA in the peptide sequence, the tool facilitates strategic decision making in developing peptides informed by the practicality of individual building block synthesis. The tool accepts the SMILES of an

NNAA as the input, protects the reactive groups, and employs a retrosynthetic planning software to generate potential synthetic routes. It then utilizes two DL-based scoring methods to assess the feasibility of the protected NNAAs through evaluating these routes. The scoring algorithms are complementary: Chemformer score validates the chemical plausibility of individual reactant–product pairs while the expert-augmented score provides an overall route feasibility assessment evaluating the proposed reaction classes as well as route-level features. The dual scoring enables more reliable route selection and NNAA prioritization. We apply retrosynthesis software and scoring models originally developed for small molecules to protected NNAAs, as they are essentially small molecules themselves.

The tool uses NNAAs enumerated from eMolecules by taking their potential precursors into account. The diverse and extensive collection of NNAAs enables the tool to cover a broad spectrum of reactive substructures, ensuring wide applicability to various NNAAs. It uses a custom heuristic, based on the literature, developed for mapping reactive groups to suitable protective groups in amino acid protection, along with a dedicated cheminformatics tool designed for this purpose. The pipeline was implemented in a modular structure, facilitating easy expansion with new mappings of the reactive fragments to protection groups.

NNAA-Synth can be applied across various peptide-related projects. In peptide library design, it supports building block selection, enabling the efficient synthesis during peptide screening for targeted research objectives. The tool also streamlines the choice of appropriate protective strategies for individual amino acids, which is critical for the directed assembly of amino acids during chemical synthesis. Furthermore, it assesses the feasibility of amino acids in peptidomimetics, guiding the optimization efforts towards practically synthesizable candidates. Integrating NNAA-Synth as a post-processing step in generative model-driven peptide design, the computer-assisted DMTA cycle can be accelerated to propose optimal peptides containing synthesizable NNAAs. While adapting to diverse demands of peptide research, our tool fundamentally evaluates the synthetic complexity of SPPS-ready NNAAs.

To illustrate our tool in practical applications, we provided two examples. The first example showed the evaluation and ranking of potential protection strategies for a specific NNAA of interest. Because the synthesis of NNAAs must be complemented by appropriate protection to maintain the efficient peptide synthesis during SPPS, the protected NNAA is regarded as the target molecule. The most feasible set of protecting groups can then be identified from the corresponding proposed routes using our tool to accomplish the synthesis of SPPS-compatible NNAAs. The second example involved a virtual screening experiment in which a set of NNAAs were docked in multiple positions of a natural peptide to optimize its binding affinity. The docking experiment was reinforced with the synthetic feasibility scores of candidate NNAAs. Selection of NNAAs driven not only by VS results but also endorsed by the synthetic feasibility considerations, can enable prioritization of mutagenesis positions as well as boosting the overall efficiency of the hit selection and the drug discovery process.

Including protection to the synthesis challenge of NNAAs addresses the true complexity of utilizing these building blocks in SPPS. Ranking the feasibility of various protection strategies can ensures NNAAs seamless integration into peptide. The future studies would focus on expanding the current tool to evaluate the factors affecting the SPPS throughput. While the tool evaluates SPPS-compatibility, it does not consider the specific reaction conditions or constraints inherent to the SPPS reaction cycle.^40^ These include amino acid instability, the influence of the reactive groups on the post-synthesis purification, and the impact on the structural integrity of peptides, such as the cyclization efficiency or intramolecular cyclization.^41^ Thus, a straightforward scoring of the entire peptide, such that the least synthesizable NNAA representing a synthetic bottleneck as the most challenging step in the overall peptide synthesis process, does not fully capture the true complexity of peptide synthesis. Additionally, the current version is limited to α-amino acids even though the current heuristics can be used for other building blocks. While the open-source solution and generalizable methodology allow for easy extension of reactive substructure-protection group mappings, incorporating specific protection strategies for other building blocks such as β, γ-amino acids, could broaden its applicability. These potential improvements require aligning the tool with the evolving landscape of peptide synthesis. This is especially important given the rapid advancements in SPPS technologies aimed at achieving higher throughput and the recent initiatives toward green chemistry with more environmentally friendly reagents.^41–43^ Another aspect is the performance of the retrosynthesis planning also depending on the reactions included in training data for both AiZynthFinder and the DL-based scoring models. Therefore, augmenting the training data with commonly used reactions for amino acid synthesis and protection could also improve the quality of the proposed routes. Although our tool does not tackle every challenge related to chemical synthesis of peptides, it represents, to our knowledge, the first computational tool for synthesizability assessment of NNAAs.

## Conclusions

In this study, we have introduced a novel comprehensive computational tool, NNAA-Synth, that integrates building block protection and synthetic planning with feasibility evaluation, specifically tailored for individual NNAAs. By providing orthogonal protection for NNAAs, it captures the synthesis requirements such as mitigating undesired side reactions during SPPS and conducts synthetic feasibility assessments on retrosynthetic routes proposed for these protected NNAAs. This synthesizability assessment can be leveraged to (i) determine the optimal protection strategies to make SPPS-compatible NNAAs, (ii) prioritize or select NNAAs in a synthesizability-informed manner. Through this synthesis assistance, NNAA-Synth contributes to enhancing the efficiency of peptide drug discovery process by facilitating the seamless transition from *in silico* design of novel NNAAs to chemical synthesis of the peptides.

## Author contributions

G. G., M. K., S. G. conceptualized the project and develop the methodology. G. G. conducted the study and wrote the manuscript. T. K. and C. K. contributed to the implementation of the heuristics and the application of AiZynthFinder. L. D. M., F. D. and O. E. provided feedback to the manuscript. All authors reviewed the manuscript.

## Conflicts of interest

There are no conflicts to declare.

## Supplementary Material

SC-016-D5SC04898B-s001

## Data Availability

The NNAA library used to build this tool can be found in Amarasinghe et al.’s supplementary files^9^ : https://pubs.acs.org/doi/suppl/10.1021/acs.jcim.2c00193/suppl_file/ci2c00193_si_001.txt. The NNAA protection and route scoring utilities have been incorporated to reaction-utils^44^ in https://github.com/MolecularAI/reaction_utils. The retrosynthetic planning tool can be accessed from https://github.com/MolecularAI/aizynthfinder. The Chemformer model checkpoint used for route scoring is available in https://zenodo.org/records/16920392. An end-to-end demo for NNAA protection, retrosynthesis, and scoring by NNAA-Synth tool is described in https://github.com/MolecularAI/nnaa_synthesizability. Supplementary information is available. See DOI: https://doi.org/10.1039/d5sc04898b.
