# Supplementary material for: From concept to chemistry: integrating protection group strategy and reaction feasibility into non-natural amino acid synthesis planning
Source: Chem Sci. 2025 Sep 8;16(38):17927–38. doi: 10.1039/d5sc04898b (PMC12415892; doi:10.1039/d5sc04898b)
Supplement: SC-016-D5SC04898B-s001 [file SC-016-D5SC04898B-s001.pdf]

Supplementary Information (SI) for Chemical Science.
This journal is © The Royal Society of Chemistry 2025

Supplementary File

From Concept to Chemistry: Integrating protection group strategy and reaction feasibility into non-natural amino acid synthesis planning

Gökçe Geylan*[ab], Mikhail Kabeshov[a], Samuel Genheden[a], Christos Kannas[a], Thierry Kogej[a], Leonardo De Maria[c], Florian David[b], Ola Engkvist[ad]

a. Molecular AI, Discovery Sciences, BioPharmaceuticals R&D, AstraZeneca, Gothenburg, Sweden.
b. Division of Systems and Synthetic Biology, Department of Life Sciences, Chalmers University of Technology, Gothenburg, Sweden.
c. Medicinal Chemistry, Research and Development, Respiratory & Immunology, BioPharmaceuticals R&D, AstraZeneca, Gothenburg, Sweden
d. Department of Computer Science and Engineering, Chalmers University of Technology and University of Gothenburg, Gothenburg, Sweden
** Email: gokcegeylan96@gmail.com*

Table of Contents

Figure S1 2
Figure S2 3
Figure S3 4
Figure S4 5
Table S1 6
Table S2 8
Table S3 9

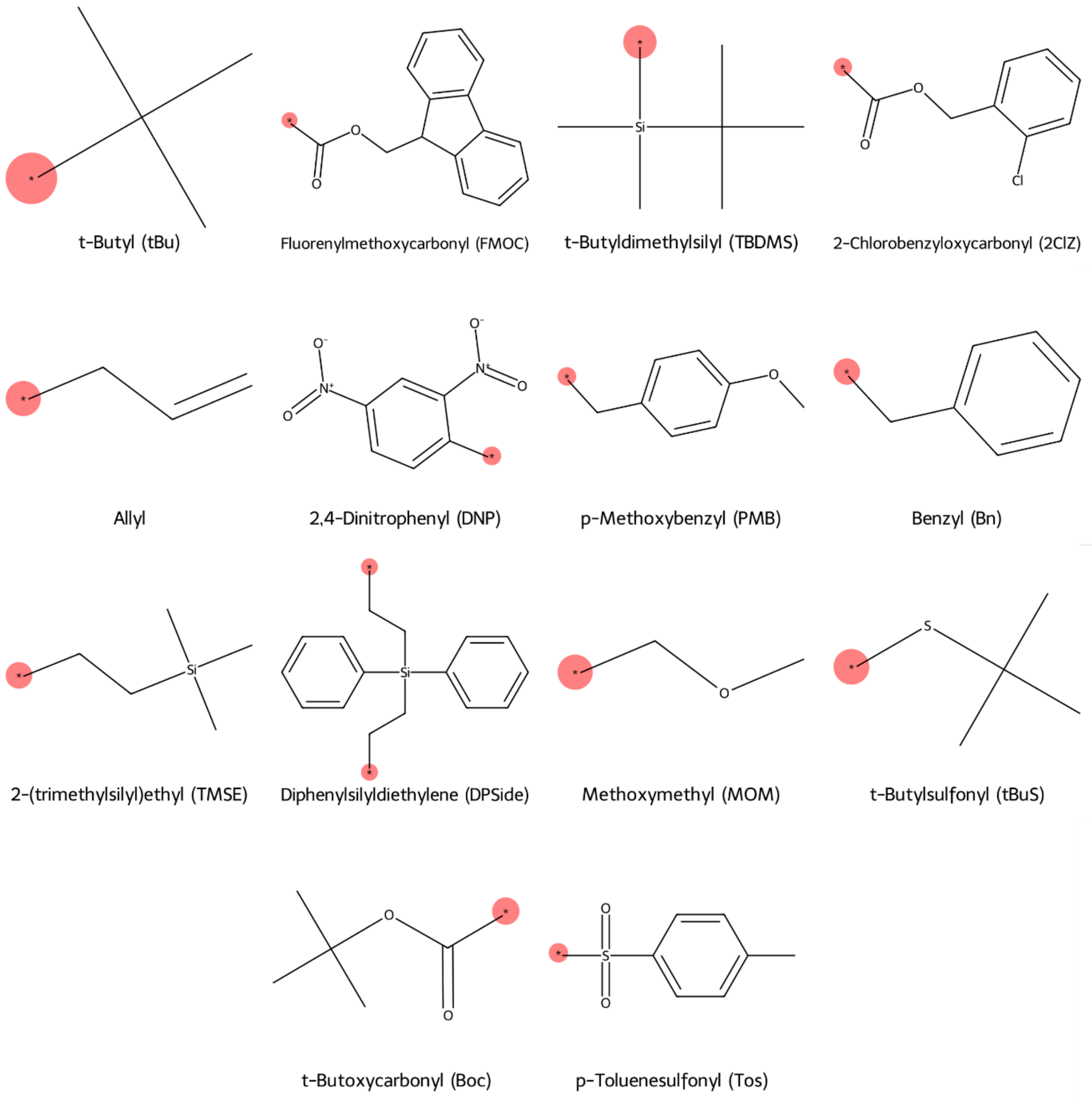

Figure S1. The structures and the connection points (in red) of the protection groups included in amino acid protection, labelled with abbreviations.

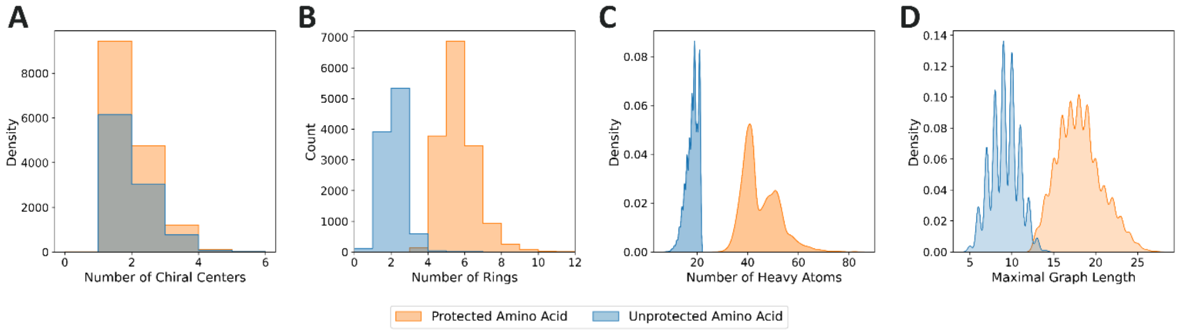

Figure S2. Molecular complexity differences of the chemical spaces defined by the protected (orange) and unprotected (blue) amino acids are illustrated. These differences are shown in terms of A) number of chiral centers, B) number of rings, C) number of heavy atoms and D) maximal graph length.

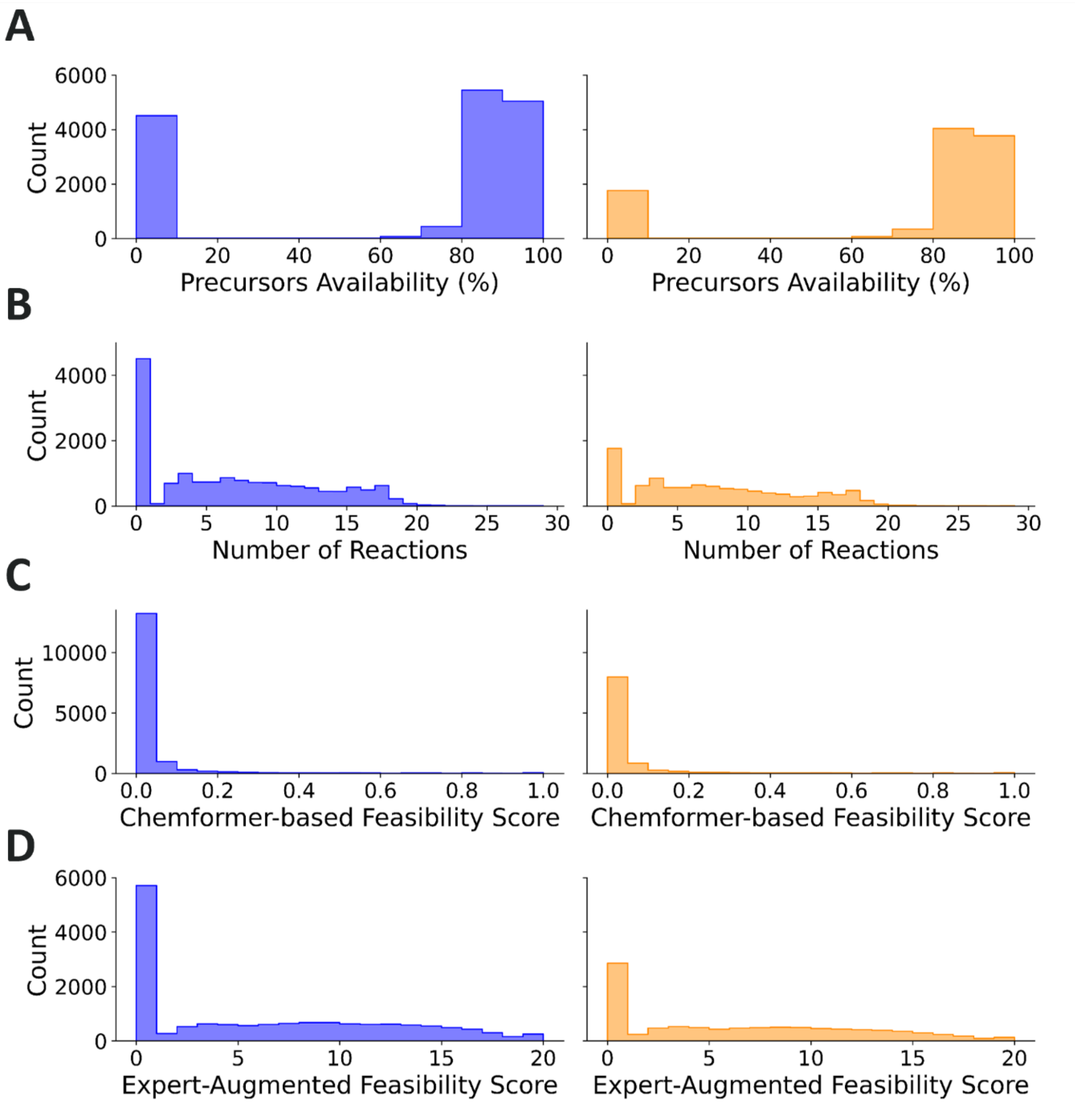

Figure S3. The overall quality-related distributions of the routes proposed by AiZynthFinder including A) the percentage of available precursors, B) number of reactions proposed for the target molecules, C) feasibility scores computed by Chemformer, and D) feasibility scores computed by the expert-augmented deep learning model. The distributions were plotted for the best route chosen for both the protected NNAAs (blue), and the individual NNAAs in the most feasible protected form (orange) as the target molecules.

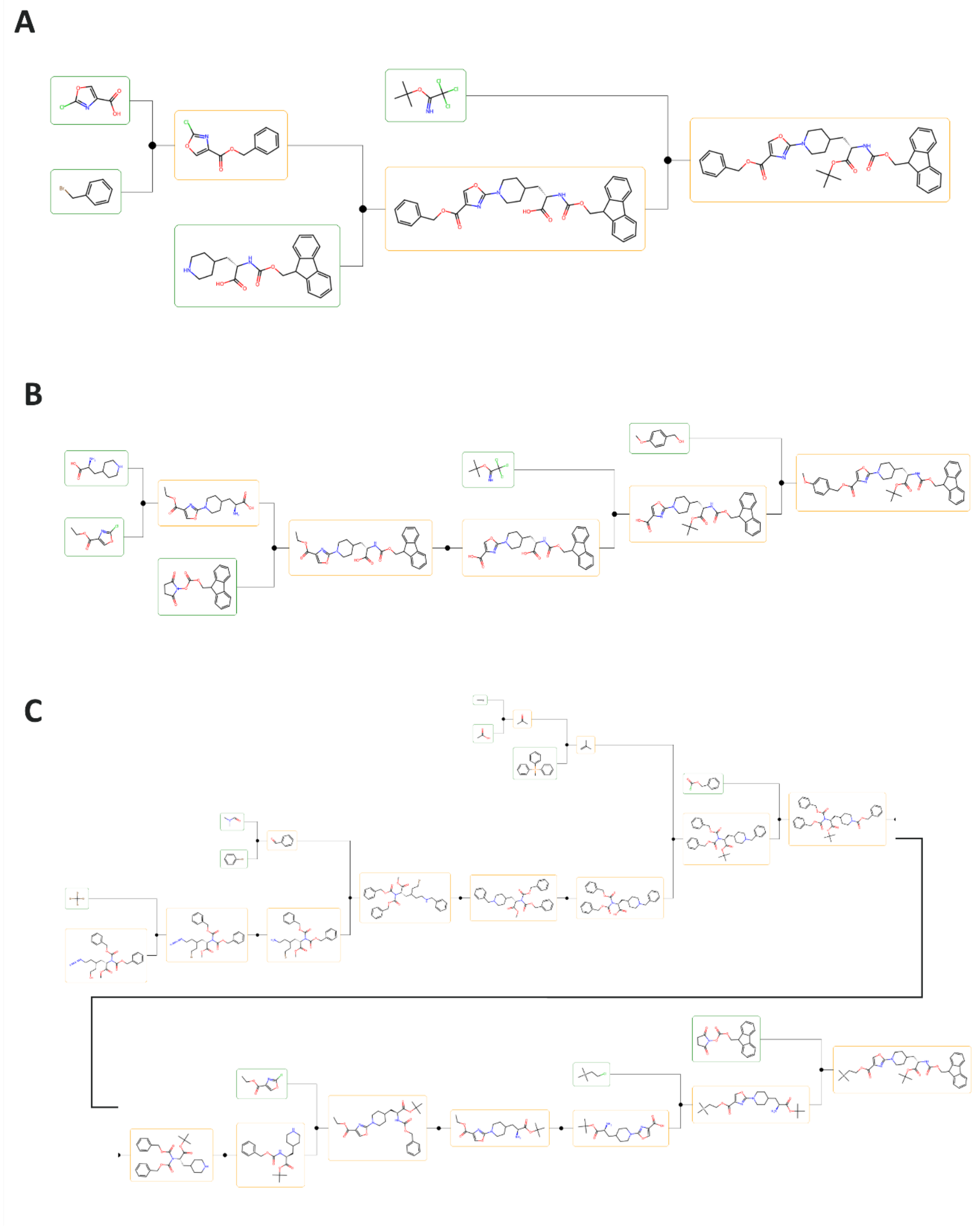

Figure S4. The proposed routes for 2G6 with protection groups of A) Fmoc, tBu, Bn with SF score of 5.12, B) Fmoc, tBu, PMB Bn with SF score of 7.13, and C) Fmoc, tBu, TMSE with SF score of 19.91. The starting materials, the proposed intermediates and the target molecule of the routes are outlined with a green frame if they are available in the provided stocks, and with a yellow frame if they are not.

Table S1. Reference SMARTS patterns for the reactive groups requiring protection. The reactive groups written in bold describe the substructures in the backbone of the α-amino acids.

Reactive Group Name	Substructure SMARTS
AcidPhenyl	[O;$([OD1]C(=O)[c;$(c1ccccc1)]):1][C;$(C(=O)O):2][c:3]
AcidPhenylThio_3	[O;$([O;D1]S(=O)[c;$(c1ccccc1)]):1][S;$(S(=O)O):2][c:3]
AcidHerero6Aromatic	[O;$([OD1]C(=O)[c;$(c1*****1);!$(c1ccccc1)]):1][C;$(C(=O)O):2][c:3]
AcidHerero5Aromatic	[O;$([O;D1]C(=O)[c;$(c1****1);!$(c1cccc1)]):1][C;$(C(=O)O):2][c:3]
**AcidAliphaticAlphaCarbon**	**[O;$([O;D1]C(=O)[A]);$(OC(=O)C[N]):1][C;$(C(=O)O):2][C:3]**
AcidAliphatic	[O;$([O;D1]C(=O)[A]);!$(OC(=O)C[ND1]):1][C;$(C(=O)O):2][C:3]
AcidAliphaticThio_3	[O;$([O;D1]S(=O)[A]):1][S;$(S(=O)O):2][C:3]
AminePhenylPrimary	[ND1;!+;$(N[c;$(c1ccccc1)]):1][c;$(cN):2]
AminePhenylSecondary	[ND2;!+;$(N[C]);$(N[c;$(c1ccccc1)]);!$(N=*);!$(N*=*):1][c;$(cN):2]
AmineHetero6AromaticPrimary	[ND1;!+;$(N[c;$(c1*****1);!$(c1ccccc1)]);!$(N*=*):1][c;$(cN):2]
AmineHetero6AromaticSecondary	[ND2;!+;$(N[C]);$(N[c;$(c1*****1);!$(c1ccccc1)]);!$(N=*);!$(N*=*):1][c;$(cN):2]
AmineHetero5AromaticPrimary	[ND1;!+;$(N[c;$(c1****1);!$(c1cccc1)]);!$(N*=*):1][c;$(cN):2]
AmineHetero5AromaticSecondary	[ND2;!+;$(N[C]);$(N[c;$(c1****1);!$(c1cccc1)]);!$(N=*);!$(N*=*):1][c;$(cN):2]
**AmineBenzylPrimaryAlphaCarbon**	**[ND1;!+;$(NCc);$(NCC(=O)O);!$(N*=*);!$(Nc):1][C;$(C([N;D1])c):2]**
AmineBenzylPrimary	[ND1;!+;$(NCc);!$(N*=*);!$(Nc);!$(NCC(=O)O):1][C;$(C([N;D1])c):2]
AmineBenzylSecondary	[ND2;!+;$(N(C)Cc);!$(N*=*);!$(N=*);!$(Nc):1][C;$(C([N;D2])c):2]
**AmineAliphaticPrimaryAlphaCarbon**	**[ND1;!+;!$(NCc);$(NCC(=O)O);!$(N*=*):1][C;$(C[N;D1]):2]**
AmineAliphaticPrimary	[ND1;!+;!$(NCc);$(N[C]);!$(N*=*);!$(NCC(=O)O):1][C;$(C[N;D1]):2]
AmineAliphaticAcyclicSecondary	[ND2;!R;!+;!$(NCc);$(N[C]);!$(NO);!$(N=*);!$(N*=*);!$(Nc):1][C;$(C[N;D2]):2]
AmineAliphaticCyclicSecondary	[ND2;R;!+;!$(NCc);$(N[C]);!$(NO);!$(N=*);!$(N*=*);!$(Nc):1][C;$(C[N;D2]):2]
AlcoholPhenyl	[O;D1;$(O[c;$(c1ccccc1)])][c;$(cO)]
ThiolPhenyl	[S;D1;$(S[c;$(c1ccccc1)])][c;$(cS)]
AlcoholHetero6Aromatic	[O;D1;$(O[c;$(c1*****1);!$(c1ccccc1)])][c;$(cO)]
ThiolHetero6Aromatic	[S;D1;$(S[c;$(c1*****1);!$(c1ccccc1)])][c;$(cS)]
AlcoholHetero5Aromatic	[O;D1;$(O[c;$(c1****1);!$(c1cccc1)])][c;$(cO)]
ThiolHetero5Aromatic	[S;D1;$(S[c;$(c1****1);!$(c1cccc1)])][c;$(cS)]
AlcoholAliphaticPrimary	[OD1;$(O[C;D2,D1]);!$(O*=*):1][C;$(CO):2]
AlcoholAliphaticSecondary	[OD1;$(O[C;H1]);!$(OC=*):1][C;$(CO):2]

AlcoholAliphaticTertiary	[OD1;$(O[C;H0]);$(OC(C)(C)C):1][C;$(CO):2]
Aromatic5nH	[n;H1;$(n1****1)]
Aromatic6nH	[n;H1;$(n1*****1)]
AmineConjugatedSecondary	[N;R;H1;D2;$(N(c)),$(N[C,c]=[N,n])]
Imine_2	[N;D1;$(N=[C;$(C[O,S,N])])]
ImineAcyclicAlcohol	[OD1;$(O([ND2]));!$(O(N(n)=C));!$(O(NOC));$(O(N=[C;!$(C[O,S,N])]))]
Amidine	[N;D1,D2;$(N[C;$(C=N);!$(C=[ND3]);!$(C(N)N)])]
Carbamic Acid	[O;$([O;D1][C;D3](N)(=O))]

Table S2. Mapping of the reactive groups to potential protection groups.

Reactive Group Name	Protection Group Names
AminePhenylSecondary	DNP, 2ClZ
AmineHetero6AromaticPrimary	2ClZ, DPSide
AlcoholAliphaticTertiary	allyl, Bn, TMSE, PMB
AmineHetero5AromaticSecondary	DNP, 2ClZ
AminePhenylPrimary	2ClZ, DPSide
AmineHetero5AromaticPrimary	2ClZ, DPSide
AcidPhenyl	allyl, Bn, TMSE, PMB
AlcoholHetero6Aromatic	allyl, Bn, TMSE, PMB
AmineBenzylSecondary	DNP, 2ClZ
AlcoholHetero5Aromatic	allyl, Bn, TMSE, PMB
AmineAliphaticAcyclicSecondary	DNP, 2ClZ, PMB, Bn
AcidHerero6Aromatic	allyl, Bn, TMSE, PMB
Amidine	DNP, 2ClZ, PMB, Bn
ThiolPhenyl	tBuS, TMSE
ImineAcyclicAlcohol	allyl, Bn, TMSE, PMB
ThiolHetero5Aromatic	tBuS, TMSE
Imine_2	Tos, 2ClZ
AcidAliphaticThio_3	TMSE, Bn, PMB
Carbamicacid	TMSE, allyl
AcidPhenylThio_3	TMSE, Bn, PMB
ThiolHetero6Aromatic	tBuS, TMSE
AcidAliphaticAlphaCarbon	tBu
AcidAliphatic	TMSE, allyl
AmineAliphaticPrimaryAlphaCarbon	Fmoc
AmineBenzylPrimaryAlphaCarbon	Fmoc
AmineAliphaticPrimary	2ClZ, DPSide
AmineBenzylPrimary	2ClZ, DPSide
Aromatic5nH	DNP, 2ClZ
AlcoholAliphaticSecondary	allyl, MOM
Aromatic6nH	DNP, 2ClZ
AmineHetero6AromaticSecondary	DNP, 2ClZ
AmineAliphaticCyclicSecondary	DNP, 2ClZ, PMB, Bn
AlcoholPhenyl	allyl, Bn, TMSE, PMB
AlcoholAliphaticPrimary	allyl, Bn, TMSE, PMB
AmineConjugatedSecondary	DNP, 2ClZ, Boc
AcidHerero5Aromatic	allyl, Bn, TMSE, PMB

Table S3. The synthetic feasibility assessments of all the docked NNAAs in three positions–Leucine, Aspartic Acid and Glutamic Acid at positions 76, 77, and 78 respectively–are shown. The docked NNAAs are categorized into "Good", "Plausible", and "Bad" feasibility groups, describing the distribution of synthetically feasible NNAAs considered for mutating each position.

Mutagenesis Position	Total Docked NNAAs	Synthetic Feasibility		
		Bad	Plausible	Good
L76	8070	2701	1509	2560
D77	4726	1382	842	1868
E78	8223	2867	1474	2554
